# Supplementary material for: Dynamic transcriptomic profiles of zebrafish gills in response to zinc supplementation
Source: BMC Genomics. 2010 Oct 11;11:553. doi: 10.1186/1471-2164-11-553 (PMC3091702; doi:10.1186/1471-2164-11-553)
Supplement: Additional file 2 — Interactive Direct Interaction Network representing the molecular interactions between zinc, copper, iron, calcium and proteins encoded by transcripts changed by zinc supplementation. Mini web-site containing index.html and hyperlinked pages in subdirectory describing a Direct Interaction Network automatically generated based on curated interactions contained within the proprietary PathwayArchitect database. Ovals represent proteins and the circles symbolize metal ions. Objects are coloured by their abundance in zebrafish at the time-point they were significantly different from the control is a scale from -4 fold (dark green) to +4 fold (dark red). Where significant differences were found at more than one time-point, the colour overlay shows expression at the first instance. Dark blue squares denote 'binding', and light blue squares 'expression'; green squares stand for 'regulation', green diamonds for 'metabolism', and green circles for 'promoter binding'. Arrow heads indicate directionality of the interaction where annotated. All nodes and edges can be further interrogated by selecting the relative area of the image. [file 1471-2164-11-553-S2.zip › PathwayArchitect Zn xs DIN/441927.html]

# REGULATION:

|  |  |
| --- | --- |
| Type | REGULATION |
| Effect | Positive |


---

|  |  |
| --- | --- |
| Score | 0 |


---

|  |  |
| --- | --- |
| Reference Count | 4 |


---

|  |  |
| --- | --- |
| Mechanism | Unknown |


---

|  |  |
| --- | --- |
| Reference:0 || Sentence | "It is 30 years since Ebashi and colleagues showed that Ca2+ ions directly affect regulation of the myosin-actin interaction in muscle through the action of tropomyosin and troponin on muscle thin filaments." |
| PMID | 9657517 |
| Year | 1998 |
| Species | Human |
| Journal | FASEB J |
| RefScore | 1 |
| Source | PArchNLP |
  |
|  |  |


---

|  |  |
| --- | --- |
 Reference:1 || Sentence | "However, Ca2+ significantly increased the accessibility of the TnI-1 epitope on TnI in the presence of troponin C and troponin T." |
| PMID | 11327886 |
| Year | 2001 |
| Species | Mouse |
|  | Human |
|  | Rat |
| Journal | Biochemistry |
| RefScore | 2 |
| Source | PArchNLP |
  ||  |  |


---

|  |  |
| --- | --- |
 Reference:2 || Sentence | "Ca(2+) binding causes structural changes between cTnC and two critical regions of cardiac muscle troponin I (cTnI): the regulatory region (cTnI-R, residues 150-165) and the inhibitory region (cTnI-I, residues130-149)." |
| PMID | 12511564 |
| Year | 2003 |
| Journal | J Biol Chem |
| RefScore | 2 |
| Source | PArchNLP |
  ||  |  |


---

|  |  |
| --- | --- |
 Reference:3 || Sentence | "In the presence of calcium ions, tissue transglutaminase catalyzes the polymerization of skeletal muscle troponin to high molecular weight insoluble aggregate." |
| PMID | 7818521 |
| Year | 1995 |
| Species | Human |
| Journal | Biochem Biophys Res Commun |
| RefScore | 0 |
| Source | PArchNLP |
  |


---

|  |  |
| --- | --- |
